# Does a History of Migraine Affect the Rate of Thrombolysis in Young Stroke Patients?

**DOI:** 10.1155/2013/351064

**Published:** 2013-11-14

**Authors:** Halvor Øygarden, Christopher Elnan Kvistad, Lars Thomassen, Ulrike Waje-Andreassen, Halvor Naess

**Affiliations:** ^1^Department of Neurology, Haukeland University Hospital, PB 1400, 5021 Bergen, Norway; ^2^Institute of Clinical Medicine, University of Bergen, PB 7800, 5020 Bergen, Norway

## Abstract

*Background*. Migraine is prevalent in young patients and a frequent stroke mimic. To distinguish stroke mimics from true stroke can be difficult, and there is a possibility of misdiagnosing a stroke as a migrainous attack in patients with migraine. We aimed to investigate if a history of migraine affects the rate of thrombolytic therapy in young stroke patients. *Methods*. All patients below 50 years of age admitted in the period 2006–2013 to the Bergen Stroke Centre with acute ischaemic stroke were included. The rate of thrombolytic therapy in patients with migraine was compared to patients with no history of migraine. A multivariate analysis was performed to adjust confounding factors. *Results*. A total of 170 young stroke patients were enrolled, 49 with migraine and 121 with no migraine. In total, 10.2% of young patients with migraine received thrombolytic therapy, compared with 26.5% of young patients with nomigraine (*P* = 0.02). Migraine was associated with a low rate of thrombolytic therapy when adjusting for possible confounding factors (OR 0.19 CI: 0.05–0.72, *P* = 0.02). *Conclusion*. Migraine is associated with a low rate of thrombolytic therapy in young patients admitted with acute ischaemic stroke. Migraine patients admitted with acute ischaemic stroke are at risk of maltreatment.

## 1. Introduction

Migraine is a common neurological disorder with a prevalence of approximately 6–15% in males and 15–25% in females [1–3]. While migraine is highly prevalent in young adults, the incidence of ischaemic stroke is relatively low (6.6 to 11.4 in 100 000 people/year) [4, 5]. In an emergency setting, it may be difficult to distinguish stroke from stroke mimics (SM) [6]. The administration of tPA to patients with SM is safe [7, 8]. It may thus be worse to refrain from treating an acute ischaemic stroke patient with tPA under suspicion of stroke mimic than to unnecessarily treat a stroke mimic with tPA.

Migraine aura is a common SM and may be misinterpreted as an ischaemic event [6–9]. Conversely, migraine patients can have ischaemic events which are misinterpreted as SM. This matter is especially crucial in emergency situations where thrombolytic therapy may be withheld in patients with a history of migraine because the treating doctor suspects SM and not ischaemic stroke. This problem is highly relevant in migraine patients since this patient group has been associated with a higher risk of ischaemic stroke [10, 11]. In this study, we aimed to examine the rate of thrombolytic therapy in young stroke patients with and without a history of migraine. We hypothesised that migraine would be associated with a lower rate of thrombolytic therapy, suggesting that tPA treatment may be withheld in acute ischaemic stroke (AIS) patients with a history of migraine. 

## 2. Methods

All stroke patients admitted to the Bergen stroke centre between February 2006 and April 2013 were prospectively registered in the NORSTROKE registry. AIS was defined in accordance with the Baltimore-Washington Cooperative Young Stroke Study Criteria comprising neurological deficits lasting longer than 24 hours or transient ischemic attacks in which computed tomography (CT) or magnetic resonance imaging (MRI) showed infarction related to symptoms [12]. In this retrospective analysis, patients with ischaemic stroke below the age of 50 years were included. A structured interview was performed by a neurologist during the hospital stay and data regarding migraine was registered. Patients were stratified in migraine and no-migraine groups according to migraine history. Baseline characteristics and definitive diagnoses were validated before inclusion. Based on presenting symptoms, patients were classified according to the Oxford Community Stroke Project classification (OCSP) [13]. Information regarding headache and aura as part of the acute symptomatology was not registered. 

Six cerebrovascular risk factors were assessed according to a predefined protocol: angina pectoris, myocardial infarction, intermittent claudication, diabetes mellitus, hypertension, and smoking. Angina pectoris, myocardial infarction, and intermittent claudication were based on diagnoses made before the index stroke. Diabetes mellitus was considered present if the patient was on glucose-lowering treatment by diet or medication. Hypertension was defined as antihypertensive treatment before the index stroke. Smoking was trichotomised in never, former, and current smoking. Current smoking was present if the patient was smoking at least one cigarette per day within 1 year before stroke onset. A risk factor score representing the number of risk factors present in each patient was given as 0, 1, 2, or >2.

### 2.1. Statistics

STATA 12.1 (StataCorp, College Station, TX) was used for all analyses. 

Student's *t*-test was used for continuous variables, Wilcoxon rank sum test was used for continuous variables not normally distributed, and Chi-square test was used for categorical variables. A logistic regression analysis with tPA administration as a dependent variable was performed in order to identify the association between a history of migraine and chance of thrombolytic therapy. Analyses were adjusted for potential confounders by stepwise backward selection. A separate univariate analysis was performed in patients admitted within 270 minutes after stroke onset, that is, patients eligible for thrombolytic therapy, to investigate the rate of tPA treatment in eligible patients in detail. To investigate the impact of age, we performed an identical logistic regression analysis including patients within the age range of 50–80 years to see if migraine influenced the rate of thrombolytic therapy in this older age group. 

The significance level was set at 0.05. 

## 3. Results

In total, 170 patients were included, 112 males and 58 females. There were 49 patients in the migraine group and 121 patients in the no-migraine group. Demographics are shown in Table 1. Median age was 43.3 years (IQR: 34.0–46.4) in the migraine group and 43.5 years (IQR: 34.4–46.4) in the no-migraine group (*P* = 0.31). There were fewer smokers (*P* = 0.02) and significantly fewer risk factors present in the migraine group (*P* < 0.01). At time of admission, there was no difference as to OCSP. The rate of MRI was similar in the two groups. The percentages of patients admitted within the therapeutic window among the migraine and no-migraine group were 30.6% versus 40.5%, respectively (*P* = 0.62).

The median NIHSS score upon admission was 2 in both the migraine (IQR: 0–8) and no-migraine group (IQR: 0–6, *P* = 0.90). Clinical data and acute management upon admission are shown in Table 2. In total, 10.2% of patients in the migraine group received thrombolytic therapy versus 26.5% in the no-migraine group (*P* = 0.02). Logistic regression analysis showed that a diagnosis of migraine (OR 0.19 CI: 0.05–0.72, *P* = 0.02) and longer time from ictus to admission (OR 0.91 CI: 0.83–0.98, *P* = 0.03) were associated with a lower rate of tPA treatment (Table 3).

Analysing only the 59 patients admitted within 270 minutes, that is, eligible for thrombolytic therapy, displayed a difference in rate of tPA treatment with 26.7% of patients in the migraine group receiving therapy compared to 59.1% in the no-migraine group (*P* = 0.03). One patient in the migraine group and one patient in the no-migraine group could not receive thrombolytic treatment due to elevated international normalized ratio (INR) (*P* = 0.57). There was no significant difference in MRI verification of AIS diagnosis (*P* = 0.16). All patients in the migraine group had diffusion lesions on MRI 24 h after admittance verifying the diagnosis of AIS, 4 of the patients in the no-migraine group were not examined by MRI, and 2 did not have diffusion lesions present, thus receiving a diagnosis of AIS on basis of symptoms persisting >24 h. Logistic regression analysis on patients admitted within the thrombolytic time window showed an association between migraine and a low rate of thrombolytic therapy (OR 0.16 CI: 0.03–0.72, *P* = 0.017). Total risk factor score, time from ictus to admission, and NIHSS on admission did not affect the rate of thrombolytic therapy (Table 3). In patients admitted within the thrombolytic time window aged 50–80 years, no association between migraine and tPA treatment was found. Higher NIHSS on admission and shorter time from ictus to admission were associated with tPA treatment (Table 4). 

## 4. Discussion

This study shows a significantly lower rate of thrombolytic therapy among young AIS patients with migraine as compared with AIS patients with no migraine. The low rate of thrombolytic therapy may result from misdiagnosing AIS symptoms as migrainous phenomena in young patients with migraine. This misdiagnosing may be apparent only in young patients due to the low incidence of stroke compared to the relatively high incidence of migrainous attacks in this age group [1, 4, 5]. The low rate may also be a result of doctors being reluctant to treat young patients with migraine and possible AIS because of the risk of intracerebral haemorrhage (ICH) after tPA administration, especially if only mild AIS symptoms are present. However, since administration of tPA in SM patients is proven safe, this should not be a sole reason to refrain from treating a possible AIS with tPA [7, 8]. The fewer risk factors present in patients with migraine may mislead the doctor, making a diagnosis of AIS appear less likely, thereby supporting the theory of misdiagnosing. This theory is also supported by the finding of no association between migraine and tPA treatment in patients aged 50–80 years. Age is an important risk factor for stroke and higher age will therefore increase the likelihood that stroke-like symptoms are caused by AIS [14, 15].

Although migraine is more common in females, there was a predominance of males in both the migraine and no-migraine groups in our material; this is a result of the inclusion criteria of acute ischaemic stroke in young adults, which is a more frequent event in males than females. Migrainous attacks are common with almost 14% of young adults experiencing a migrainous attack in one year [3]. A Danish study investigating prevalence and incidence rates of migrainous aura reported a 1–3 year prevalence of 4.1 and an incidence rate of 5.6 per 1000 person-years [16]. This indicates that migrainous aura may be a common phenomenon in young adults, making the task of differentiating migraine aura from AIS a frequent task for the doctor treating stroke patients. 

The present study highlights that a correct diagnosis of AIS is imperative to ensure that neither tPA treatment is wrongly withheld from AIS patients on suspicion of SM nor patients with SM are treated with tPA unnecessarily. Clinical predictors for improved diagnostic accuracy have been identified, but their usefulness in clinical practice is uncertain [17, 18]. Increased use of imaging modalities such as CT perfusion or MR diffusion weighted imaging in the emergency department can improve the diagnostic accuracy and minimise the risk of clinical misinterpretation of AIS and possible maltreatment of young patients with migraine [18–20]. 

There are some limitations in this study. The sample size is relatively small, even after 6 years of inclusion. Secondly, since only hospitalized AIS patients were eligible for inclusion, patients with possible AIS not admitted to the hospital were therefore not included in our study; this could be a source of selection bias. However, the Bergen Stroke Centre enforces a zero threshold for admitting patients with symptoms indicating stroke; we therefore believe that the vast majority of patients were admitted and therefore eligible for inclusion. This is supported by the low median NIHSS scores. The low NIHSS scores also indicate that small ischaemic strokes with minor deficits are included; this can however not explain the observed low rate of treatment in patients with migraine since these minor strokes are equally distributed between the two groups. There was neither a significant difference in stroke severity as to NIHSS on admission when comparing migraine and no-migraine patients receiving tPA nor was there any difference when comparing the migraine and no-migraine patients not receiving tPA. 

In conclusion, migraine is associated with a low rate of thrombolytic therapy in young patients admitted with acute ischaemic stroke. Migraine patients with acute ischaemic stroke may therefore run the risk of maltreatment and poor clinical outcome. 

## Figures and Tables

**Table 1:** Demographic characteristics of young ischaemic stroke patients with and without migraine.

	Migraine	No-migraine	*P*
	*N* = 49	*N* = 121	
Median age, years (IQR)	43.3 (34.0–46.4)	43.5 (38.0–46.4)	0.31
Male sex (%)	57.1	69.4	0.12
Smoking status (%)			0.03
Never smoked	55.1	40.2	
Former smoker	24.5	17.9	
Current smoker	20.4	41.9	
Hypertension (%)	18.4	26.5	0.26
Diabetes (%)	6.1	6.7	0.90
Hypercholesterolemia (%)	4.1	10.2	0.13
Atrial fibrillation (%)	0	5.0	0.11
Prior VD (%)	8.1	12.6	0.41
Total risk factors (%)			<0.01
0	69.4	37.2	
1	18.4	43.8	
2	8.2	12.4	
>2	4.1	6.6	

IQR: interquartile range; VD: vascular disease (defined as prior cerebrovascular disease, cardiovascular disease, or peripheral vascular disease).

**Table 2:** Clinical characteristics and initial management of young ischaemic stroke patients with and without migraine.

	Migraine	No-migraine	*P*
	*N* = 49	*N* = 121	
Median NIHSS score (IQR)	2 (0–8)	2 (0–6)	0.90
Median ictus to admittance time (min., IQR)	191 (96–336)	135 (70–511)	0.55
Presenting symptomatology (OCSP)			0.75
LACI (%)	18.4	22.3	
TACI (%)	8.2	10.7	
PACI (%)	44.9	36.4	
POCI (%)	28.6	30.6	
Admitted < 270 min after ictus (%)	30.6	40.5	0.86
CT-scan as initial imaging (%)	77.6	77.7	0.99
Receiving thrombolytic therapy (%)	10.2	26.5	0.02

NIHSS: National Institute of Health Stroke Scale; IQR: interquartile range; OCSP: Oxford Community Stroke Project Classification; LACI: lacunar infarct; TACI: total anterior circulation infarct; PACI: partial anterior circulation infarct; POCI: posterior circulation infarct; CT: computed tomography.

**Table 3:** Logistic regression analysis displaying factors associated with thrombolytic therapy (rTPA) in all young AIS patients and young AIS patients admitted within the thrombolytic time window.

	All patients			Admitted within 270 min		
	OR	95% CI	P	OR	95% CI	P
Migraine	0.19	0.05–0.72	0.02	0.16	0.04–0.72	0.02
Time from ictus to admission in hours	0.90	0.83–0.98	0.02	0.94	0.84–1.06	0.32
NIHSS on admission	1.09	0.99–1.18	0.05	1.07	0.97–1.17	0.17
Total risk factor score	0.56	0.27–1.17	0.12	0.47	0.19–1.13	0.09

AIS: acute ischaemic stroke; NIHSS: National Institutes of Health Stroke Scale; OR: odds ratio; CI: confidence interval.

There was an association between migraine and a low rate of received rTPA treatment in young patients with acute ischaemic stroke.

**Table 4:** Logistic regression analysis displaying factors associated with thrombolytic therapy (tPA) in AIS patients admitted within the thrombolytic time window between 50 and 80 years of age.

	OR	95% CI	*P*
Migraine	1.48	0.84–2.61	0.17
Time from ictus to admission in hours	0.92	0.86–0.97	<0.01
NIHSS on admission	1.07	1.13–1.25	<0.01
Total risk factor score	0.94	0.74–1.17	0.57

AIS: acute ischaemic stroke; NIHSS: National Institutes of Health Stroke Scale; OR: odds ratio; CI: confidence interval.

There was an association between shorter time from ictus to admission, higher NIHSS on admission, and high rate of rTPA treatment in acute ischaemic stroke patients between 50 and 80 years of age.
